# A Novel Biomimetic Nanosponge Protects the Retina from the *Enterococcus faecalis* Cytolysin

**DOI:** 10.1128/mSphere.00335-17

**Published:** 2017-11-22

**Authors:** Austin L. LaGrow, Phillip S. Coburn, Frederick C. Miller, Craig Land, Salai Madhumathi Parkunan, Brian T. Luk, Weiwei Gao, Liangfang Zhang, Michelle C. Callegan

**Affiliations:** a Department of Ophthalmology, University of Oklahoma Health Sciences Center, Oklahoma City, Oklahoma, USA; b Department of Cell Biology, University of Oklahoma Health Sciences Center, Oklahoma City, Oklahoma, USA; c Department of Family and Preventive Medicine, University of Oklahoma Health Sciences Center, Oklahoma City, Oklahoma, USA; d Department of Microbiology and Immunology, University of Oklahoma Health Sciences Center, Oklahoma City, Oklahoma, USA; e Dean McGee Eye Institute, Oklahoma City, Oklahoma, USA; f Department of NanoEngineering and Moores Cancer Center, University of California San Diego, San Diego, California, USA; University at Buffalo

**Keywords:** *Enterococcus*, cytolysin, cytotoxins, endophthalmitis, eye infection, nanoparticle, nanosponge

## Abstract

Intraocular infections are a potentially blinding complication of common ocular surgeries and traumatic eye injuries. Bacterial toxins synthesized in the eye can damage intraocular tissue, often resulting in poor visual outcomes. *Enteroccocus faecalis* causes blinding infections and is responsible for 8 to 17% of postoperative endophthalmitis cases. These infections are increasingly difficult to treat due to the emergence of multidrug-resistant strains. Virulent *E. faecalis* isolates secrete a pore-forming bicomponent cytolysin that contributes to retinal tissue damage during endophthalmitis. We hypothesized that a biomimetic nanosponge, which mimics erythrocytes, might adsorb subunits of the cytolysin and reduce retinal damage, protecting vision. To test the efficacy of nanosponges in neutralizing the cytolysin *in vitro*, hemoglobin release assays were performed on culture supernatants from cytolysin-producing *E. faecalis* with and without preincubation with nanosponges. Treatment with nanosponges for 30 min reduced hemolytic activity by ~70%. To determine whether nanosponges could neutralize the cytolysin *in vivo*, electroretinography was performed on mice 24 h after intravitreal injection with cytolysin-containing supernatants treated with nanosponges. Pretreatment of cytolysin-containing supernatants with nanosponges increased the A-wave retention from 12.2% to 65.5% and increased the B-wave retention from 21.0% to 77.0%. Histology revealed that in nanosponge-treated eyes, retinas remained intact and attached, with little to no damage. Rabbit nanosponges were also nontoxic and noninflammatory when injected into mouse eyes. In an experimental murine model of *E. faecalis* endophthalmitis, injection of nanosponges into the vitreous 6 h after infection with a wild-type cytolysin-producing strain increased A-wave retention from 5.9% to 31% and increased B-wave retention from 12.6% to 27.8%. Together, these results demonstrated that biomimetic nanosponges neutralized cytolysin activity and protected the retinas from damage. These results suggest that this novel strategy might also protect eyes from the activities of pore-forming toxins of other virulent ocular bacterial pathogens.

**IMPORTANCE** Endophthalmitis is a serious, potentially blinding infection that can result in vision loss, leaving a patient with only the ability to count fingers, or it may require enucleation of the globe. The incidence of postoperative endophthalmitis has markedly increased over the past 2 decades, paralleling the rise in ocular surgeries and intravitreal therapies. *E. faecalis* is a leading cause of infection following ocular procedures, and such infections are increasingly difficult to treat due to multidrug resistance. Cytolysin is the primary virulence factor responsible for retinal tissue damage in *E. faecalis* eye infections. Treatment of these infections with antibiotics alone does not impede ocular damage and loss of visual function. Pore-forming toxins (PFTs) have been established as major virulence factors in endophthalmitis caused by several bacterial species. These facts establish a critical need for a novel therapy to neutralize bacterial PFTs such as cytolysin. Here, we demonstrate that biomimetic nanosponges neutralize cytolysin, protect the retina, preserve vision, and may provide an adjunct detoxification therapy for bacterial infections.

## Abstract (precis)

Endophthalmitis is a serious, potentially blinding infection that can result in vision loss, leaving a patient with only the ability to count fingers, or it may require enucleation of the globe. The incidence of postoperative endophthalmitis has markedly increased over the past 2 decades, paralleling the rise in ocular surgeries and intravitreal therapies. *E. faecalis* is a leading cause of infection following ocular procedures, and such infections are increasingly difficult to treat due to multidrug resistance. Cytolysin is the primary virulence factor responsible for retinal tissue damage in *E. faecalis* eye infections. Treatment of these infections with antibiotics alone does not impede ocular damage and loss of visual function. Pore-forming toxins (PFTs) have been established as major virulence factors in endophthalmitis caused by several bacterial species. These facts establish a critical need for a novel therapy to neutralize bacterial PFTs such as cytolysin. Here, we demonstrate that biomimetic nanosponges neutralize cytolysin, protect the retina, preserve vision, and may provide an adjunct detoxification therapy for bacterial infections.

## INTRODUCTION

Intraocular infections are often devastating and result in vision loss and blindness. Intraocular infection (also termed endophthalmitis) occurs as a result of the introduction of microorganisms into the eye. The most frequent type occurs during or following a surgical procedure (postoperative endophthalmitis [POE]). Surgeries, such as those to treat cataracts or glaucoma, and ocular injections to treat degenerative diseases, such as age-related macular degeneration (AMD), risk introducing microorganisms from surrounding tissue or contaminated surgical instruments into a normally sterile and immune-privileged environment. Ocular surgeries and intraocular injections have dramatically increased over the last several decades, and this in turn has been correlated with an increase in the incidence of POE (1–6). In 70% of severe cases of POE, the final visual acuity is worse than 20/100 (7). Endophthalmitis can also occur during or after a traumatic penetrating injury to the globe (posttraumatic endophthalmitis [PTE]). While PTE is not as frequent as POE, the rate of infection is higher, ranging from 3 to 17%, and the visual outcome is often worse after a penetrating injury to the eye than following a surgical procedure (8, 9). Endogenous endophthalmitis (EE) occurs following hematogenous spread of microorganisms from a distant focal infection in the body into the eye. EE is usually associated with an underlying medical condition, such as diabetes mellitus, a compromised immune system, or intravenous drug abuse (1–3, 8, 10–12). The visual prognosis following EE is uniformly poor, with a reported median final visual acuity of 20/100 (13).

Current treatments for intraocular infections include intravitreal and systemic administration of antibiotics and the surgical removal of the vitreous humor of the eye. However, once symptoms are present irreversible damage to nonregenerative tissues of the eye may have already occurred due to toxin production by the infectious agent and the host inflammatory response. The time from intraocular bacterial contamination of the eye to the discovery of symptoms and initiation of treatment is often several hours. As such, despite antibiotic and anti-inflammatory treatment, infections with virulent pathogens often lead to poor visual outcomes that range from only being able to count fingers to complete blindness (1–3, 8, 10–12). In the most severe cases, infected eyes may be enucleated. This potentially devastating result indicates the need for new therapeutic agents, as the current treatment regimens do not target or do not affect the events that lead to vision loss.

Bacterial pore-forming toxins (PFTs) are key factors for retinal tissue damage in intraocular infections (14–20). PFTs are the largest group of bacterial virulence factors, comprising approximately 25 to 30% of bacterial cytotoxic proteins (21), and include *Staphylococcus aureus* alpha-toxin, *Streptococcus pneumoniae* pneumolysin, and *Enterococcus faecalis* cytolysin (14–20). PFTs are logical targets for therapies aimed to reduce toxin-mediated damage, given their demonstrable roles in endophthalmitis pathogenesis. A number of anti-PFT neutralization strategies have been developed, including anti-PFT antibody and vaccine approaches (22–25). While antibody-based PFT neutralization methods have proven effective at reducing disease severity and lethality in mouse models, antibodies targeting specific PFTs lack broad applicability. Hu et al. developed a biomimetic nanosponge that binds and neutralizes broad-spectrum bacterial PFTs regardless of their molecular structures (26). Nanosponges consist of a polymeric nanoparticle core surrounded by a natural red blood cell membrane. Nanosponges act by presenting themselves as a decoy and irreversibly binding PFTs, thus preventing them from acting on their normal target cells. Nanosponges administered before or after a subcutaneous injection with *S. aureus* alpha-toxin effectively protected mice from developing edema, inflammation, and skin lesions. In addition, the systemic administration of nanosponges markedly reduced mortality rates from a lethal dose of alpha-toxin (26). Alpha-toxin is a key virulence factor in rabbit and mouse models of endophthalmitis (18, 27), so nanosponges might have applicability as a novel therapeutic agent for intraocular infections caused by alpha-toxin-producing strains of *S. aureus*. Escajadillo et al. demonstrated that local administration of nanosponges successfully neutralized streptolysin O, a pore-forming toxin and key virulence factor produced by *Streptococcus pyogenes*, and effectively reduced the severity of an *S. pyogenes* necrotizing skin infection in a murine model (28). The *S. pneumoniae* pneumolysin also contributes to endophthalmitis pathogenesis, and a vaccine approach was effective in neutralizing pneumolysin in the eye (29). The *E. faecalis* cytolysin is a PFT and primary virulence factor in endophthalmitis. No studies have been published on strategies for neutralization of cytolysin as a means to attenuate enterococcal disease in general, or in the eye specifically. Because cytolysin is the only PFT secreted by *E. faecalis*, we sought to test the ability of nanosponges to neutralize cytolysin as a proof-of-concept model for nanosponge-based PFT neutralization strategies aimed at reducing intraocular damage and vision loss.

**FIG 1:**
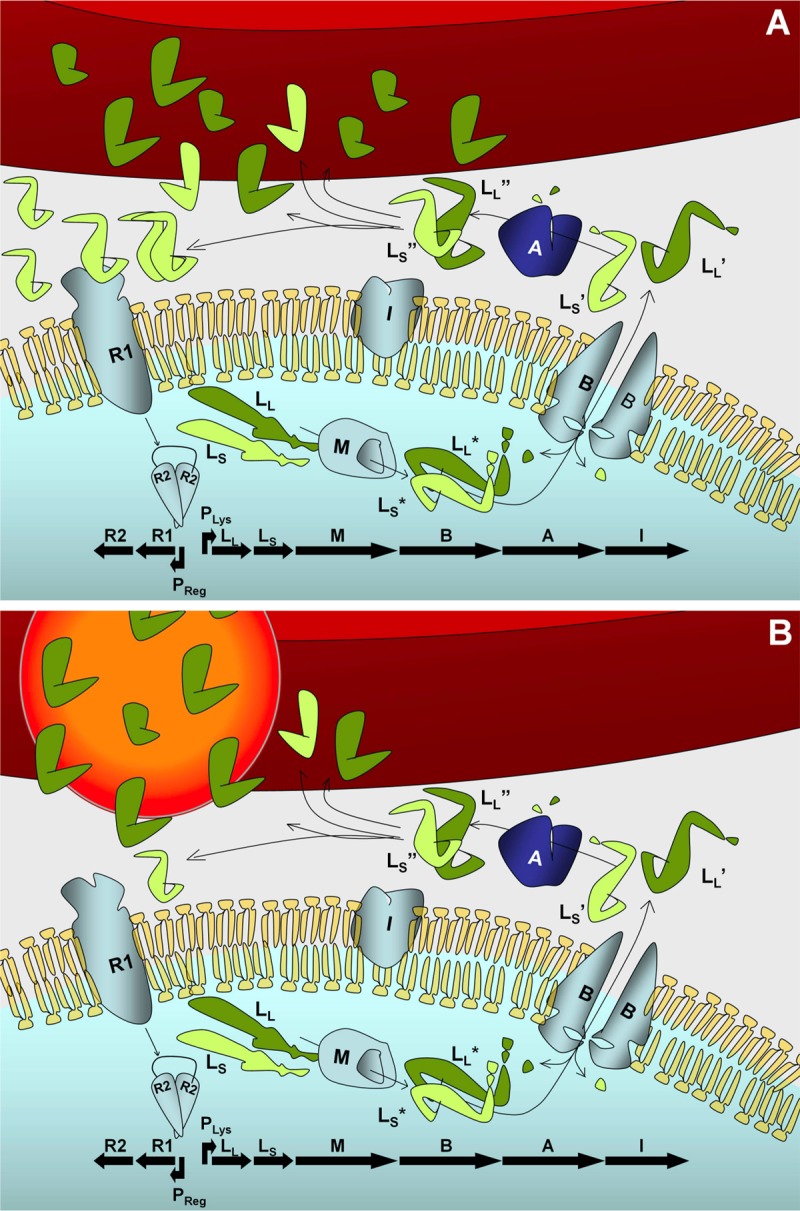
Model of the *E. faecalis* cytolysin and its neutralization by nanosponges. (A) CylL_L_ and CylL_S_ are ribosomally synthesized and posttranslationally modified by CylM, generating CylL_L_* and CylL_S_*. After modification, both subunits are secreted and processed by CylB, generating CylL_L_′ and CylL_S_′. Following secretion, both subunits are further processed by CylA, generating the active toxin subunits CylL_L_″ and CylL_S_″. The large subunit, CylL_L_″, has a greater affinity for the target cell membrane than CylL_S_″, which in the presence of a target cell is believed to result in transient accumulation of excess free CylL_S_″, generating a quorum-sensing autoinduction signal that triggers release of CylR2 and high-level expression of the cytolysin operon. CylL_L_″ and CylL_S_″ subunits coordinate to form a pore in the target membrane and cause target cell lysis (11, 12). (B) Nanosponges might interfere with membrane pore formation by selectively interacting with CylL_L_″ and prevent binding to the target cell and subsequent interaction with CylL_S_″.

*E. faecalis* is a health care-associated pathogen that is among the leading causes of nosocomial infections (30). *E. faecalis* has acquired resistance to the majority of available therapeutic agents and is ranked seventh among the CDC’s top antibiotic-resistant threats (31). *E. faecalis* is also a leading cause of POE, particularly following glaucoma surgery. Highly virulent strains of *E. faecalis* express a single PFT termed cytolysin. The active cytolysin consists of large (CylL_L_″) and small (CylL_S_″) peptide subunits (Fig. 1). Both subunits are required for cytotoxic activity. The CylL_L_″ subunit has a significantly higher affinity for erythrocytes than CylL_S_″ and binds preferentially to target erythrocytes (32, 33). CylL_S_″ is then presumably recruited into a multimeric complex that forms a pore in the target cell. Cytolysin, in addition to being the only PFT secreted by *E. faecalis*, has a demonstrable role in the pathogenesis of enterococcal endophthalmitis in a rabbit model. Stevens et al. established that cytolysin contributed to significant damage of the neuroretinal architecture of the eye (34). In this model, anti-inflammatory and antibiotic drugs did not attenuate the infection or improve retinal function retention. However, using the same model and treatment strategy against an isogenic noncytolytic strain completely attenuated the infection. That study demonstrated the importance of cytolysin as a primary virulence factor in *E. faecalis* endophthalmitis, as well as the need to develop a novel therapy option for targeting the cytolysin in intraocular infections when more traditional therapies do not work.

The present study investigates whether a biomimetic nanosponge protects the eye from the detrimental effects of *E. faecalis* cytolysin. We hypothesized that nanosponges will prevent retinal tissue damage in a murine model of sterile, cytolysin-induced endophthalmitis by irreversibly binding to and neutralizing the CylL_L_″ cytolysin subunit. Our results demonstrate that nanosponges neutralize the CylL_L_″ subunit and reduce the hemolytic activity of the *E. faecalis* cytolysin. In both sterile *in vivo* and live organism endophthalmitis models, nanosponges reduce damage to the architecture of the eye and preserve retinal function. This study establishes nanosponges as a novel and potentially feasible approach to targeting the *E. faecalis* cytolysin, a significant contributor to retinal toxicity during intraocular infection.

(This work was presented in part at the ASM Microbe 2017 meeting in New Orleans, LA.)

## RESULTS

### Nanosponges reduced cytolysin-mediated hemolysis *in vitro*.

**FIG 2:**
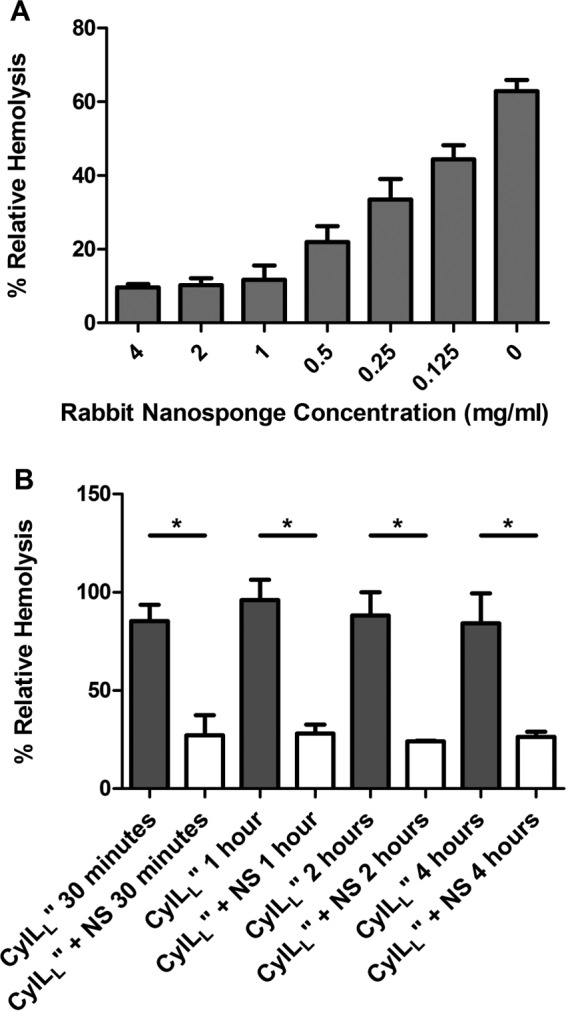
Nanosponges reduced cytolysin hemolytic activity *in vitro*. (A) Undiluted filter-sterilized supernatant from an 18-h culture of a CylL_L_″-producing strain, FA2-2 (pWH851), was mixed 1:1 with various nanosponge (NS) concentrations ranging from 8 mg/ml to 0.25 mg/ml, such that the final concentrations ranged from 4 mg/ml to 0.125 mg/ml, or with PBS, and allowed to incubate at 37°C for 30 min. Nanosponges were removed by centrifugation, and hemolytic activity was assessed as described in Materials and Methods. Values represent mean results ± SEM of three independent experiments. Significance was set at a *P* value of <0.0001. (B) Undiluted filter-sterilized supernatant from an 18-h culture of the CylL_L_″-producing strain FA2-2 (pWH851) was mixed 1:1 with a solution of 8 mg/ml nanosponges, such that the final concentration was 4 mg/ml, or with PBS and allowed to incubate at 37°C for either 30 min or 1, 2, or 4 h. Nanosponges were removed by centrifugation, and hemolytic activity was assessed as described in Materials and Methods. Values represent mean results ± SEM of three independent experiments. *, P ≤ 0.0029.

To test the efficacy of the biomimetic nanosponges derived from rabbit erythrocytes to neutralize cytolytic activity of *E. faecalis* and to optimize the nanosponge concentration and neutralization time, we performed hemolysis assays on sterile culture supernatants from previously generated *E. faecalis* strains that produced either CylL_L_″ or CylL_S_″. *E. faecalis* strain FA2-2 (pWH851) produces only CylL_L_″, while the isogenic strain FA2-2 (pWH617) produces only CylL_S_″ (32). As shown in Fig. 2A, preincubation of CylL_L_″-containing supernatant with increased concentrations of nanosponges for 30 min resulted in decreased levels of hemolytic activity (*P* < 0.0001). These results suggested that nanosponges bind to CylL_L_″ to inhibit hemolysis. A nanosponge concentration of 8 mg/ml (final concentration of 4 mg/ml after mixing 1:1 with sterile supernatant) was shown to be a sufficient concentration of nanosponges to provide maximal reduction of hemolytic activity (Fig. 2A). After mixing CylL_L_″ supernatants 1:1 with 8 mg/ml nanosponges or phosphate-buffered saline (PBS), significant differences between nanosponge-treated CylL_L_″ and untreated CylL_L_″ supernatants were observed for 30 min, 1 h, 2 h, and 4 h of preincuation (*P* ≤ 0.0029) (Fig. 2B). Nanosponges reduced hemolytic activity to a similar degree regardless of the time of incubation with CylL_L_″-containing supernatant, indicating that saturation of nanosponges occurred in ≤30 min (*P* = 0.8436) (Fig. 2B). Since 30 min was a sufficient amount of time for maximum neutralization of CylL_L_″, this length of time was used for our incubation period throughout the remainder of the *in vitro* and *in vivo* experiments.

### Nanosponge neutralization of CylL_L_″ protected retinal function.

**FIG 3:**
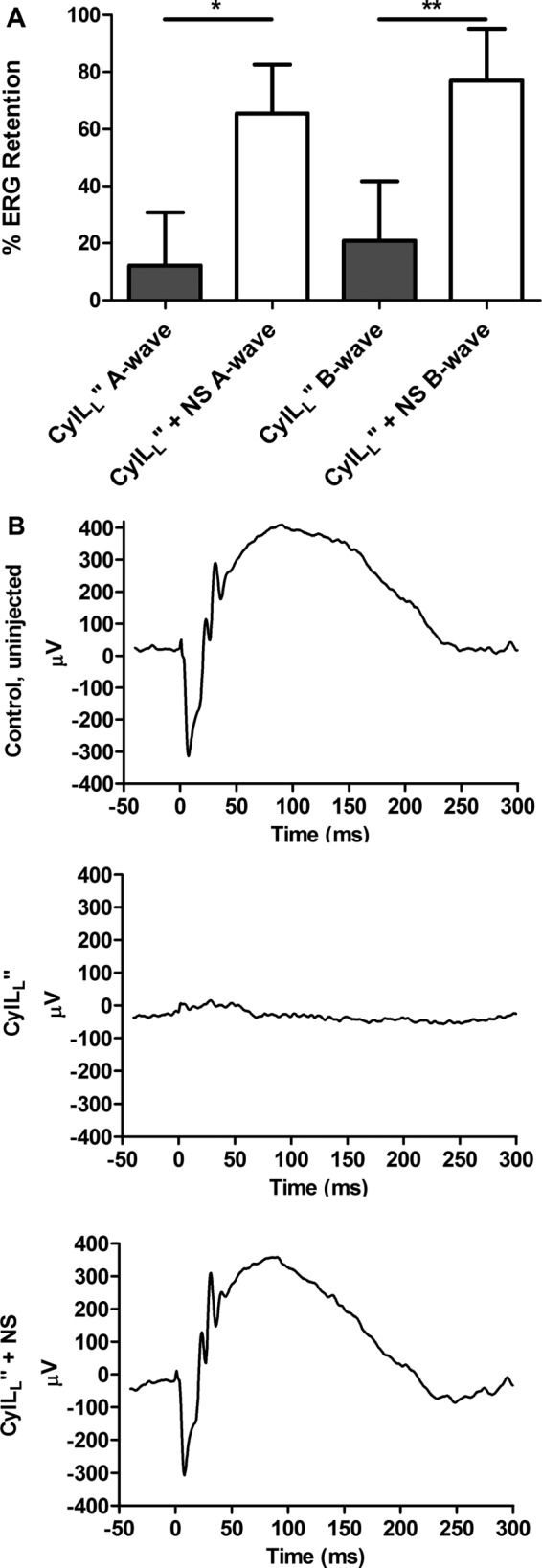
Nanosponge neutralization of CylL_L_″ improved retinal function retention. Eyes of C57BL/6J mice were injected with either 0.5 µl of nanosponge-treated CylL_L_″ supernatant (NS) or untreated CylL_L_″ supernatant, followed immediately by injection of 0.5 µl of CylL_s_″ supernatant. Retinal function was assessed by electroretinography 24 h postinjection. (A) Eyes injected with nanosponge-treated CylL_L_″ supernatant and CylL_S_″ supernatant had significantly higher A-wave retention (*, *P* = 0.0000029) and B-wave retention (**, *P* = 0.0000048) than eyes injected with untreated CylL_L_″ supernatant plus CylL_S_″ supernatant. Values represent mean results ± SEM of 10 eyes per group in two independent experiments. (B) Representative waveforms of control, uninjected eyes, eyes injected with untreated CylL_L_″ supernatant plus CylL_s_″ supernatant or with nanosponge-treated CylL_L_″ supernatant plus CylL_s_″ supernatant.

Electroretinography (ERG) of mouse eyes injected with nanosponge-treated CylL_L_″ supernatant and CylL_S_″ supernatant revealed significantly higher retention than after injection with untreated CylL_L_″ supernatant plus CylLS″ supernatant (Fig. 3). Eyes injected with untreated CylL_L_″ supernatant plus CylL_S_″ supernatant had a mean A-wave retention of 12.2% and a mean B-wave retention of 20.8%. However, eyes injected with nanosponge-treated CylL_L_″ supernatant and CylL_S_″ supernatant had a mean A-wave retention of 65.5% (*P* = 2.9 × 10^−6^ versus untreated CylL_L_″) and a mean B-wave retention of 76.9% (*P* = 4.8 × 10^−6^ versus untreated CylL_L_″) (Fig. 3). These results demonstrated that the rabbit nanosponges effectively neutralized the CylL_L_″ subunit, resulting in significantly greater retinal function retention *in vivo*.

### Nanosponge neutralization of CylL_L_″ protected retinal architecture.

**FIG 4:**
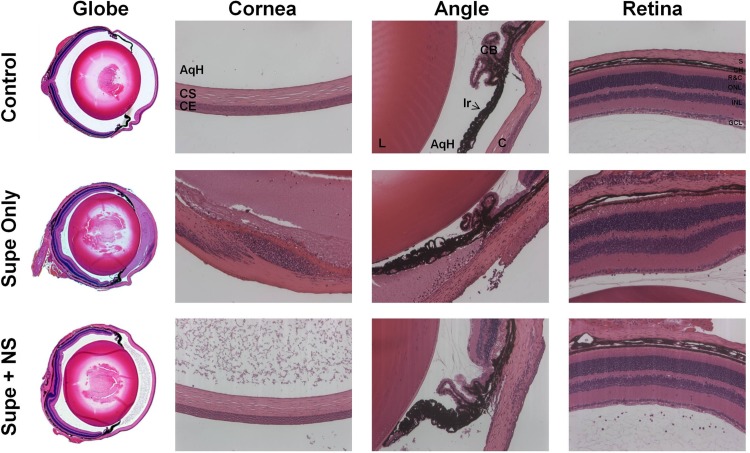
Nanosponges neutralized CylL_L_″ and protected retinas from cytolysin-mediated damage. Eyes were injected with either 0.5 µl of nanosponge-treated CylL_L_″ supernatant or untreated CylL_L_″ supernatant, followed immediately by injection of 0.5 µl of CylL_S_″ supernatant. Eyes were then harvested 24 h later and processed for hematoxylin and eosin staining. Images are representative of at least 3 eyes from at least 2 independent experiments. Uninjected C57BL/6J eyes showed no inflammation and were architecturally intact. Eyes injected with untreated CylL_L_″ supernatant showed retinal edema and swelling, the cornea was edematous with cellular infiltrate emanating from the limbus, and the anterior chamber was filled with fibrinous exudate. In contrast, in eyes injected with nanosponge-treated CylL_L_″ supernatant, histology showed no retinal edema and significantly less fibrinous exudate, and corneas appeared normal.

Histology with hematoxylin and eosin staining was performed on uninjected control eyes and on eyes injected with either nanosponge-treated CylL_L_″ supernatant or untreated CylL_L_″ supernatant, followed immediately by injection of CylL_S_″ supernatant. Control, uninjected eyes showed no inflammatory infiltrate in either the anterior or posterior segments, and the retinal layers were structurally intact (Fig. 4). Mouse eyes injected with untreated CylL_L_″ showed retinal and corneal edema, cellular infiltrate into the cornea emanating from the limbus, fibrinous exudate in the anterior chamber, and cellular infiltrate and fibrinous exudate in the posterior segment (Fig. 4). However, in mouse eyes injected with nanosponge-treated CylL_L_″ followed by injection of CylL_S_″, less anterior segment infiltrate and fibrin deposition was observed, the cornea appeared normal, and no retinal edema was observed (Fig. 4). Corneal and retinal structures were similar to those of control, uninjected eyes. Taken together with the ERG data, these results demonstrated that nanosponges effectively reduced damage to the architecture of the eye and improved retinal function retention compared to eyes injected with untreated supernatants.

### Rabbit erythrocyte-derived nanosponges were not toxic to the mouse cornea or retina.

**FIG 5:**
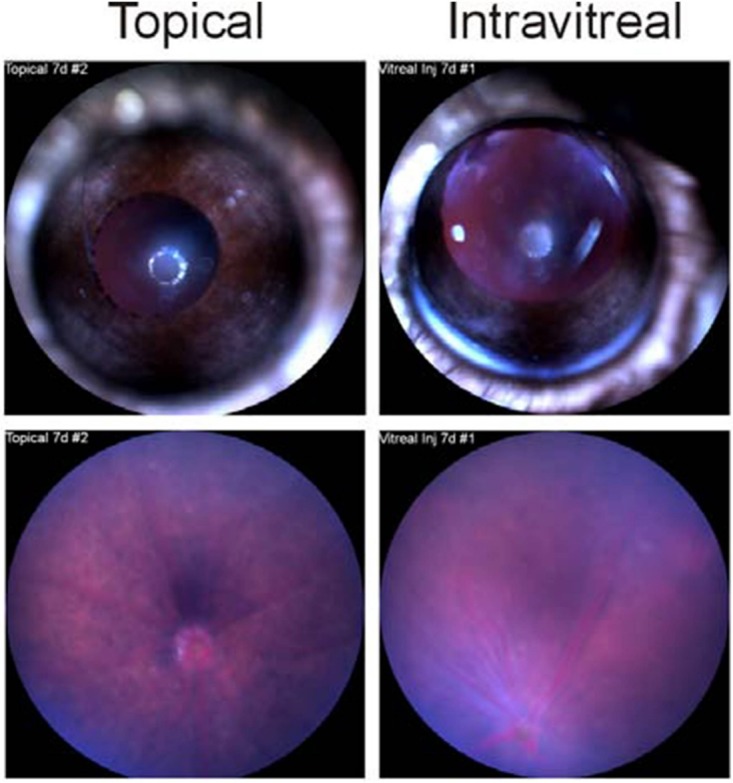
Nanosponges were not toxic to mouse eyes. Nanosponges (4 mg/ml) were topically applied or intravitreally injected into mouse eyes. Photographs and fundoscopy images were obtained at 7 days postapplication (representative results are shown for 2 eyes). Eyes treated with topical nanosponges were clear on day 7. Slight inflammation was observed in the cornea and posterior segment of intravitreally injected eyes on day 7.

Biomicroscopy and fundoscopy of rabbit nanosponge-treated eyes demonstrated no corneal or intraocular toxicity 7 days after application (Fig. 5). In eyes treated topically with nanosponges, corneas were clear and there were no apparent signs of cellular infiltrate in the aqueous humor. Fundoscopy of these eyes showed no posterior segment inflammation and a clear visual tract. In eyes treated intravitreally with rabbit nanosponges, slight inflammation was noted in the cornea and moderate inflammation was noted in the posterior segment on day 7. Fundoscopy showed some perivascular sheathing around a few of the larger retinal vessels, suggestive of a mild vasculitis. However, ERG of these eyes demonstrated no retinal function loss on day 7 after nanosponge intravitreal injection (data not shown). These results suggested that while injection of rabbit nanosponges into the mouse eye caused some inflammation, the inflammation was not as significant as that of an active infection and retinal function loss did not occur. In addition, topical administration of rabbit nanosponges to the mouse eye was also relatively safe.

### Nanosponges improved retinal function following infection with a cytolysin-producing strain of *E. faecalis*.

**FIG 6:**
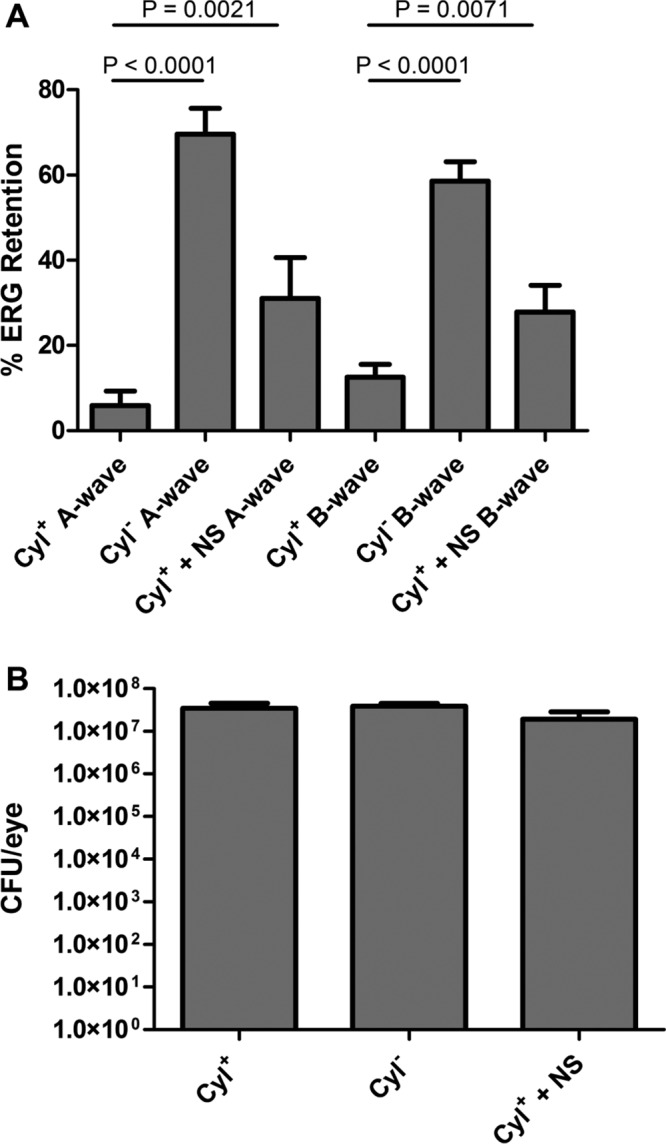
Nanosponges improve retinal function retention following infection with a cytolysin-producing strain of *Enterococus faecalis* in a murine model of endophthalmitis. Right eyes of mice were infected with 100 CFU of Cyl^+^ [FA2-2 (pAM714)] or Cyl^−^ [FA2-2 (pAM771)] strains of *Enterococcus faecalis*. At 6 h postinfection, Cyl^+^-infected right eyes were injected with 0.5 µl of 8 mg/ml (2 µg) of nanosponges. Retinal function was assessed by electroretinography at 24 h postinjection. (A) Eyes injected with Cyl^+^ *E. faecalis* showed significantly lower A-wave retention (*P* < 0.0001) and B-wave retention (*P* < 0.0001) than eyes injected with a Cyl^−^ strain of *E. faecalis*. Eyes that were treated with nanosponges 6 h after infection with a Cyl^+^ strain of *E. faecalis* demonstrated significantly improved A-wave (*P* = 0.0021) and B-wave (*P* = 0.0071) retention compared to eyes infected with a Cyl^+^ strain but did not receive nanosponge treatment. Values represent mean results ± SEM for at least 10 eyes per group in two independent experiments. (B) Eyes were harvested from mice, and *E. faecalis* counts were determined. No significant differences were observed in *E. faecalis* growth between any of the groups (*P* ≥ 0.09). Values represent mean results ± SEM of at least 8 eyes per group in two independent experiments.

Eyes infected with the wild-type, cytolytic (Cyl^+^) *E. faecalis* strain demonstrated a mean A-wave retention of 5.9%, compared to a 69.5% A-wave retention after infection with the isogenic, noncytolytic (Cyl^−^) *E. faecalis* strain 24 h after infection (*P* < 0.0001) (Fig. 6A). B-wave retention for the Cyl^+^ strain was 12.6%, and for the Cyl^−^ strain it was 58.6% (*P* < 0.0001). This result demonstrated that the cytolysin is primarily responsible for the retinal function loss after infection. However, treatment of the Cyl^+^-infected eyes with nanosponges 6 h following infection increased the A-wave retention to 31% (*P* = 0.0021) (Fig. 6A). A similar result was observed with B-wave retention, with 12.6% and 27.8% retention in untreated and nanosonge-treated mice, respectively (*P* = 0.0071) (Fig. 6A). Bacterial counts were performed on eyes from each group to ensure that the results were due to nanosponge treatment and not variations in the intraocular growth of *E. faecalis* strains. The mean concentrations of bacteria were 3.45× 10^7^/eye in untreated mouse eyes infected with the Cyl^+^ strain, 3.91× 10^7^/eye in untreated mouse eyes infected with the Cyl^−^ strain, and 1.92× 10^7^/eye in nanosponge-injected mouse eyes infected with the Cyl^+^ strain. There were no significant differences between these groups (*P* ≥ 0.09). These results indicated that comparable levels of growth of these strains occurred in the eyes of infected mice; therefore, differences in ERG retention can be attributed to cytolysin production and neutralization by nanosponges *in vivo*.

## DISCUSSION

The visual prognosis of postoperative endophthalmitis due to *E. faecalis* is uniformly poor. In the Endophthalmitis Vitrectomy Study, no patients with *E. faecalis* endophthalmitis achieved visual acuity of ≥20/40, 14.3% achieved ≥20/100 acuity, and 57.1% achieved ≥5/200 acuity (12). In a separate study of patients with *E. faecalis* endophthalmitis, 48.3% of patients achieved a final visual acuity raning from light perception to no light perception (35). *E. faecalis* accounts for 4 to 21% of cases of POE and is a frequent cause of filtering bleb infection following glaucoma surgery, which results from the introduction of organisms into the conjunctival filtering bleb following a trabeculectomy (36). The frequency of this type of endophthalmitis has been reported to be as high as approximately 10% of glaucoma filtering procedures and continues to rise with the increase in use of the antifibrotic agents (36–39). *E. faecalis* is particularly problematic in eye infections and other types of infections due to the emergence of resistance to virtually all clinically available antibiotics (30). In 2013, the Centers for Disease Control and Prevention published a report listing vancomycin-resistant enterococci as number 7 among the top 18 drug-resistant threats to the United States (31). *E. faecalis* currently accounts for 65% to 80% of all enterococcal health care-associated infections (40, 41), with *Enterococcus faecium* accounting for the majority of the remaining infection isolates.

Because of the increasing threat of multidrug-resistant infections, development of novel therapeutic treatments against *E. faecalis* infections is vital. The bicomponent cytolysin has been demonstrated to be a key contributor to *E. faecalis* virulence in multiple models of infection, from *Caenorhabditis elegans* to rabbit models (15, 34, 42–45). The *E. faecalis* cytolysin is important to pathogenesis because its activities have been shown to enhance the virulence of *E. faecalis* in animal infection models, and in epidemiological studies it has been associated with significant patient mortality (15, 30, 34, 42–46). The cytolytic phenotype is common among infection-derived isolates of *E. faecalis*, particularly those that cause hospital ward outbreaks. Studies have reported as many as 60% of infection-derived *E. faecalis* isolates to be hemolytic, compared to 17% of stool specimens from healthy volunteers (47). Other studies have reported the cytolysin determinant significantly more frequently in bacteremia isolates (34 of 68 [50%]) than in stool strains (0 of 14 [0%]) (48). In a study of a hospital ward outbreak of multiple antibiotic-resistant *E. faecalis* isolates (46), one particular genetic lineage that was both high-level gentamicin/kanamycin resistant and cytolytic caused a disproportionate number of bacteremias and deaths. Patients infected with these cytolytic, resistant strains were at a 5-fold-increased risk of death (of patients dying within 3 weeks of culture, 71% were infected with a cytolytic strain) (46), irrespective of therapy.

The cytolysin is an important mediator of damage and pathogenesis in rabbit models of *E. faecalis* endophthalmitis. Genomic fingerprinting studies of *E. faecalis* endophthalmitis isolates have shown an enrichment of the cytolysin among these strains, suggesting a potential role in endophthalmitis (27). In a rabbit model of experimental endophthalmitis, Jett and colleagues found that the cytolysin significantly contributed to the course and severity of disease and that the cytolysin was directly toxic to retinal cells (14, 15, 34). The results of the present study suggest that nanosponges may serve as an adjunct therapy, reducing cytolysin-mediated damage to the retina by interfering with the ability of one or both of the cytolysin subunits’ abilities to organize and form a membrane pore complex. In our studies, cytolysin-induced sterile endophthalmitis functioned as an appropriate model for testing the effectiveness of nanosponges in neutralizing bacterial PFTs and protecting the retina. The *E. faecalis* cytolysin displays toxin activity against cells from mammals to invertebrates, suggesting that cytolysin targets a highly conserved feature of the eukaryotic cellular membrane. The toxin is active against human, bovine, equine, and rabbit erythrocytes (49). The active cytolysin consists of two nonidentical, posttranslationally modified lytic peptides, both of which are necessary for cytotoxicity. Coburn et al. previously determined that the larger of the two peptides, CylL_L_″, binds with higher affinity to target cell membranes than the smaller subunit, CylL_S_″ (Fig. 1A) (32). Using surface plasmon resonance, Coburn and colleagues determined that the mean dissociation constant (*K*_*D*_) of CylL_L_″ for liposomes composed of phosphatidylcholine-cholesterol was 5.9 μM, and for CylL_S_″ the *K*_*D*_ was 38.1 μM (32). The CylL_L_″ subunit binds to phosphatidylcholine-cholesterol lipid bilayers with 6.5-fold-greater affinity than does CylL_S_″. Based on these studies, we reasoned that nanosponges might preferentially bind to the CylL_L_″ subunit and prevent association with target erythrocytes. Since both subunits are required to affect target cell lysis (32, 33), nanosponge-mediated reduction of the concentration of CylL_L_″ was predicted to reduce hemolysis of erythrocytes. When CylL_L_″-containing *E. faecalis* supernatant was incubated with an equal volume of 8 mg/ml nanosponges (800 μg) for 30 min, we observed a significant decrease in hemolytic activity after adding CylL_S_″-containing supernatant (Fig. 2), indicating that inhibition of the cytolysin occurred via nanosponges binding to CylL_L_″. Furthermore, incubation of CylL_L_″-containing supernatant with nanosponges for 1, 2, and 4 h did not further reduce hemolytic activity, indicating that nanosponges were effectively saturated at 30 min. Our previous experience in purifying the cytolysin subunits from culture supernatants revealed that under the growth conditions utilized in the current study, CylL_L_″ reaches an approximate concentration of 0.17 μg/ml and CylL_S_″ reaches a concentration of 0.18 μg/ml (32, 33). This indicates that 800 μg of nanosponges neutralizes approximately 17 ng of CylL_L_″. Hu et al. demonstrated that after the same amount of time (30 min), a 4-fold-smaller amount of nanosponges (200 µg) was effective at complete neutralization of 1.2 to 9 µg of the *S. aureus* PFT alpha-toxin (26). However, when the amount of alpha-toxin was increased to 30 µg, no reduction in hemolytic activity was observed relative to that with untreated alpha-toxin, indicating that the binding capacity of 200 µg of nanosponges was exceeded at that level of alpha-toxin (26).

Hu et al. demonstrated that nanosponges effectively neutralize the *S. aureus* PFT alpha-toxin, preventing tissue necrosis (26). Hu et al. postulated that the natural red blood cell (RBC) vesicle coating of the nanoparticle would act as an effective decoy to neutralize a wide range of PFTs and that the poly(lactic-co-glycolic acid) (PLGA) polymeric core stabilizes the vesicle to ensure an optimal half-life *in vivo*. Pretreatment of *S. aureus* alpha-toxin with nanosponges prevented tissue damage after subcutaneous injection into mice. Histologic examination revealed no microscopic tissue damage. In contrast, when injected subcutaneously without nanosponge treatment, alpha-toxin induced marked edema, inflammation, and severe skin lesions. Histologic examination further showed necrotic tissue, muscle tissue damage, and inflammation (26). These results suggested that pretreatment of *S. aureus* supernatants containing alpha-toxin might be similarly effective at reducing retinal damage following injection in the eye. However, *S. aureus* secretes a number of other toxins, including beta-, gamma-, and delta-toxins and the Panton-Valentine leukocidin (PVL) (29, 49, 50), which may directly cause structural damage to tissues in the eye or, in the case of PVL, have either anti- or proinflammatory effects. The presence of these additional virulence factors might confound the results of experiments designed to assess the effectiveness of neutralizing alpha-toxin in either a live *S. aureus* or sterile endophthalmitis model. The feasibility of this strategy for *S. aureus* ocular infections is being analyzed.

In the current study, we observed pathological changes to retinal layers 24 h after intravitreal injection of cytolysin-containing supernatants. Similarly, Stephens et al. observed damage to all retinal layers in rabbit eyes infected with a cytolysin-producing strain of *E. faecalis* (34). Loss of vision was most rapid, and sequelae of infection were most severe in rabbits infected with the cytolytic strain. Those authors observed a 48.0% ± 4.7% loss in B-wave amplitude at 24 h and a 98.3% ± 1.0% loss in B-wave amplitude by 72 h (34). We demonstrated that injection of preformed cytolysin resulted in approximately 79% loss in B-wave amplitude after 24 h, considerably worse than what Stephens et al. observed at 24 h when they injected live bacteria (34). This difference may have been due to differences between production of cytolysin in brain heart infusion (BHI) medium and in the rabbit eye during infection. However, this is unlikely to be the case, given that we saw similarly low A- and B-wave retention levels following infection with the Cyl^+^ strain as those following injection of preformed cytolysin. This suggests that the mouse retina might be highly sensitive to the effects of the cytolysin. Nevertheless, pretreatment of CylL_L_″-containing supernatant with nanosponges resulted in significantly greater B-wave amplitude retention (77%), which is comparable to the B-wave retention observed in rabbit eyes injected with an isogenic noncytolytic strain of *E. faecalis* (84.9%) 24 h after infection (34). Importantly, direct injection of rabbit nanosponges 6 h following infection with the Cyl^+^ strain resulted in significant protection, increasing the A-wave retention from 5.9% to 31% and B-wave retention from 12.6% to 27.8%. Of considerable importance and relevance to our current study is that combined antibiotic and anti-inflammatory therapies salvaged visual function in eyes infected with the isogenic noncytolytic mutant, but this combined therapy did not alter the destructive course of infection in eyes infected with the cytolytic strain (14). Given that a significant number of *E. faecalis* endophthalmitis isolates produce the cytolysin (46.4%) (51), our results suggest that adding nanosponges to the current therapeutic strategy may offer direct neutralization of toxins not targeted by antibiotics or anti-inflammatory drugs, thus improving the outcome of disease. More specifically, these results highlight the need for therapies targeting bacterial toxins produced in the eye during intraocular infections.

In addition to direct retinal toxicity, it has been hypothesized that the cytolysin might target innate inflammatory cells and may influence the host response. Polymorphonuclear leukocytes (PMNs), the primary innate immune effector cell in acute endophthalmitis, are of critical importance in the clearance of bacteria from the eye (16–18, 52, 53). Bacterial toxins function as virulence factors not only by direct tissue damage but also by modulating the innate immune response by killing PMNs or altering their function. *Staphylococcus aureus* PVL induces rapid cell death in human PMNs (19), and the *Streptococcus pyogenes* streptolysin S (SLS) inhibits PMN recruitment to the infection site (20). Miyazaki et al. (54) showed that, in addition to cytolytic activity against erythrocytes, cytolytic strains of *E. faecalis* killed mouse PMNs and macrophages. However, it is currently unknown as to whether this toxicity might serve as an immune evasion mechanism *in vivo* and allow *E. faecalis* to persist in the eye during infection.

Studies are currently in progress to assess the efficacy of nanosponges in neutralizing other PFTs from the leading causes of bacterial endophthalmitis, including *Bacillus cereus*, *Staphylococcus aureus*, and *Streptococcus pneumoniae*. Bacterial PFTs have been shown to be a key factor for retinal tissue damage in cases of intraocular infections with each of these organisms. Callegan et al. demonstrated that supernatants from *B. cereus* and *S. aureus* cultures are responsible for retinal damage and for inducing an inflammatory response in a rabbit model of endophthalmitis (16). Mutant derivatives of *S. aureus* lacking either alpha- or beta-toxin did significantly less damage to the retina than the parental strain in a rabbit model of endophthalmitis (18). Relative to an infection with 5,000 CFU of *S. aureus*, injection of 100 ng of purified alpha-toxin caused mild retinal damage and edema 24 h postinjection. This amount of toxin resulted in a decline in the A-wave ERG response of approximately 35%, compared to a 60% decline following *S. aureus* infection (27). In the *B. cereus* rabbit endophthalmitis model, insertional inactivation of the gene encoding the global regulator of *B. cereus* PFTs, *plcR*, resulted in significant attenuation of the rate of progression of disease. Retinal function in this model was completely lost by 18 h postinfection, but in the absence of the PlcR global regulator, the same extent of damage to the retina was not reached until 36 h postinfection. Similar results were achieved by mutation of the *S. aureus* global regulators Agr and Sar (20). Sanders et al. demonstrated that rabbits immunized with a form of pneumolysin that only retains 1% of its hemolytic activity prior to infection with a clinical isolate of *S. pneumoniae* significantly reduced retinal damage and improved slit lamp examination scores relative to results in mock-immunized rabbits (29). Alpha-toxin is important to the virulence of *S. aureus* in a rabbit model of keratitis (55), and chemical inhibition of alpha-toxin with a combination of cyclodextrin and cholesterol improved outcomes in rabbits with *S. aureus* corneal infections (56). Together, these studies validate the importance of a novel therapeutic option that is capable of targeting a widely diverse population of bacterial PFTs.

The results of this study demonstrate that nanosponges are capable of neutralizing the *E. faecalis* cytolysin and attenuating cytolysin-mediated damage to the mouse retina. The treatment of bacterial endophthalmitis can be complicated by the production of a myriad of PFTs, depending on the infecting organism. By functioning as decoys that capture the PFTs before they can bind to a host target cell, nanosponges could potentially neutralize a variety of PFTs, despite their diversity, and act as a novel detoxification therapy. The broad-spectrum activity of nanosponges may offer benefits in treatment before the offending organism is identified. As nanosponges apparently inflict little to no damage to the retina themselves, nanosponges may be beneficial to administer prophylactically during ocular treatments or surgeries that risk introducing bacteria into the immune-privileged environment of the eye. Our results also show that introduction of nanosponges derived from a heterologous species into the mouse eye does not elicit a significant immune response directed toward the heterologous proteins within the rabbit erythrocyte membranes. This is important in considering the use of cross-species nanosponges as potential therapeutic agents. Future studies to determine the clearance rate of the nanosponges from the eye as well as their other pharmacokinetic properties are necessary to better establish nanosponges as a novel adjunct treatment for bacterial endophthalmitis.

## MATERIALS AND METHODS

### Bacterial strains and nanosponges.

To generate culture supernatants containing either CylL_L_″ or CylL_S_″, we utilized previously generated *E. faecalis* strains that produce one or the other of each cytolysin subunit (32). Strains FA2-2 (pWH851) (CylL_L_″) and FA2-2 (pWH617) (CylL_S_″) were grown in BHI medium at 37°C for 18 h (32). Cultures were centrifuged for 10 min at 4,300 × *g*, and the supernatant was filter sterilized through a 0.22-μm Millex GP filter unit (Merck Millipore Ltd., Tullagreen, Ireland). Supernatants were kept on ice prior to *in vitro* and *in vivo* assays. *E. faecalis* strain FA2-2 (pAM714) (Cyl^+^) and the isogenic cytolysin-negative strain FA2-2 (pAM771) (Cyl^−^) were cultured in BHI medium at 37°C for 18 h prior to use in infecting mouse eyes (45).

Rabbit RBC nanosponges were prepared by a previously reported protocol. Briefly, to prepare polymeric cores, 10 ml of PLGA polymer (carboxyl acid terminated; 0.67 dl/g; 50:50 monomer ratio; 20 mg/ml in acetone; Lactel absorbable polymers) was added to 20 ml of Tris-HCl buffer (10 mM, pH 8). The solution was stirred and allowed to evaporate for 2 h. For membrane coating, purified rabbit RBC membranes were first mixed with PLGA cores at a protein-to-polymer weight ratio of 1:4, followed by sonication in a Fisher FS30D bath sonicator for 10 min. Size and zeta potential of the RBC nanosponges were measured by dynamic light scattering using a Malvern ZEN 3600 Zetasizer (26). Rabbit nanosponges had similar physicochemical properties as those of mouse RBC nanosponges, including a comparable size of about 90 nm in diameter and a surface zeta potential of −35 mV.

### *In vitro* hemolysis assays.

To determine an optimal concentration of nanosponges for cytolysin activity reduction, undiluted filter-sterilized supernatant from an 18-h culture of the CylL_L_″-producing strain, FA2-2 (pWH851), was mixed 1:1 with various nanosponge concentrations, ranging from 8 mg/ml to 0.25 mg/ml, such that the final concentrations ranged from 4 mg/ml to 0.125 mg/ml, or the supernatant was mixed with PBS (pH 7.4), and mixtures were allowed to incubate at 37°C for 30 min. Nanosponges were removed by centrifugation, and hemolytic activity was assessed by incubating the nanosponge-treated and untreated control CylL_L_″ supernatants with 5% washed rabbit erythrocytes for 30 min at 37°C to allow adherence of CylL_L_″ to the erythrocytes. After this period, an equal volume of filter-sterilized supernatant from an 18-h culture of the CylL_S_″-producing strain, FA2-2 (pWH617), was added, and hemolysis was allowed to proceed for 30 min at 37°C. Unlysed erythrocytes were removed by centrifugation at 500 × *g* for 5 min. Hemoglobin release was measured spectrophotometrically at 490 nm by using a FLUOstar Omega microplate spectrophotometer (BMG Labtech, Cary, NC). Values are expressed as the percent hemolysis relative to a 100% lysis control in which 5% rabbit erythrocytes were lysed in double-distilled H_2_O (ddH_2_O). Values represent the mean results ± standard errors of the means (SEM) of three independent experiments.

To identify an optimal time for incubation of nanosponges with CylL_L_″-containing supernatant, undiluted filter-sterilized supernatant from an 18-h culture of the CylL_L_″-producing strain, FA2-2 (pWH851), was mixed 1:1 with a solution of 8 mg/ml nanosponges or an equal volume of PBS (pH 7.4) and allowed to incubate at 37°C for either 30 min or 1, 2, or 4 h. The nanosponges were then removed via centrifugation for 5 min at 3,000 × *g*, and hemolytic assays were run for each time interval on treated or untreated supernatants. Hemolytic activity was assessed exactly as described for the dose-response analysis. Values are expressed as the percent hemolysis relative to a 100% lysis control in which 5% rabbit erythrocytes were lysed in ddH_2_O. Values represent the mean results ± SEM of three independent experiments.

### *In vivo* cytolysin-mediated retinal damage model.

This study was carried out in strict accordance with the recommendations in the *Guide for the Care and Use of Laboratory Animals* of the National Research Council (57). The protocol was approved by the Institutional Animal Care and Use Committee of the University of Oklahoma Health Sciences Center (protocol 15-103). Six-week-old C57BL/6J mice were acquired from The Jackson Laboratory (catalogue number 000664; Bar Harbor, ME). Mice were allowed to adjust to conventional housing for 2 weeks prior to injection to equilibrate their microbiota. All mice were housed under microisolation conditions on a 12-h on/12-h off light cycle prior to the experiments and then under biosafety level 2 conditions during experiments. Mice were 8 to 10 weeks of age at the time of the experiment.

Mice were anesthetized with a combination of ketamine (85 mg/kg of body weight; Ketathesia; Henry Schein Animal Health, Dublin, OH) and xylazine (14 mg/kg; AnaSed; Akorn Inc., Decatur, IL). Intravitreal injections were performed with sterile borosilicate glass micropipettes (Kimble Glass Inc., Vineland, NJ) beveled to an approximate bore size of 10 to 20 μm (BV-10 KT Brown type micropipette beveller; Sutter Instrument Co., Novato, CA, USA). Under stereomicroscopic visualization, the micropipettes were inserted just posterior to the superior limbus, and 0.5-µl volumes were injected directly into the midvitreous. Injection rates and volumes were monitored by using a programmable cell microinjector (Microdata Instruments, Plainfield, NJ). To assess nanosponge efficacy at neutralizing the cytolysin in a sterile endophthalmitis model, the right eyes of the mice were intravitreally injected with either 0.5 µl of nanosponge-treated CylL_L_″ supernatant or untreated CylL_L_″ supernatant. This was immediately followed by injection of 0.5 µl of CylL_S_″ supernatant. Left eyes served as uninjected controls. For the live *E. faecalis* endophthalmitis model, 100 CFU in 0.5 µl of either the Cyl^+^ or Cyl^−^ strain was injected into the right eyes of mice. At 6 h postinfection, 0.5 µl of 8 mg/ml (2 µg) was injected into the right eyes of the Cyl^−^-infected mice. Ocular changes were quantified via the analyses described below.

### Scotopic ERG.

Mice were dark adapted for 24 h, anesthetized, and then their eyes were dilated with topical phenylephrine. Topical anesthetic (0.5% proparacaine-HCl) was instilled in each eye prior to ERG. Gold wire electrodes were placed on the cornea of each eye, and reference electrodes were attached to the head and tail of the mouse. A series of five white light flashes were delivered to the mouse 60 s apart (10-ms duration) in order to provoke a retinal response. ERG measured A-wave function (corresponding to photoreceptor cell activity) and B-wave function (corresponding to Müller, bipolar, and amacrine cell activity). Scotopic A- and B-wave amplitudes were recorded for each eye (Espion E2; DiagnoSys, LLC, Lowell, MA). The percentage of retinal function retained in the infected eye was calculated in comparison with uninfected left eye controls as follows: 100 − {[1 − (experimental A- or B-wave amplitude)/(control A- or B-wave amplitude)] × 100}. Values represent the mean results ± SEM for at least 10 animals. Two independent experiments were performed.

### Fundoscopy.

Nanosponges at a concentration of 4 mg/ml (2 mice/route) were tested for topical and intravitreal toxicity. Five microliters was topically applied or 0.5 µl was intravitreally injected into C57BL/6J eyes. Biomicroscopy was conducted as previously described (58) at 7 days after application or intravitreal injection. For fundoscopy, mice were anesthetized as described above and imaged at 7 days after application or injection by using a Micron IV retinal imaging system (Phoenix Research Laboratories, Inc., Pleasanton, CA). Images are representative of at least 2 eyes per time point.

### Thin-section histology.

Eyes were harvested 24 h after injection of either 0.5 µl of nanosponge-treated CylL_L_″ supernatant or untreated CylL_L_″ supernatant, followed immediately by injection of 0.5 µl of CylL_S_″ supernatant. Harvested eyes were incubated in buffered zinc formalin or Davidson’s fixative for 24 h at room temperature (52, 53). Eyes were then transferred to 70% ethanol, embedded in paraffin, sectioned, and stained with hematoxylin and eosin. Images are representative of at least 3 eyes from at 2 independent experiments.

### Bacterial quantitation.

Eyes were enucleated, placed into separate tubes containing 400 μl of sterile PBS and 1.0-mm sterile glass beads (BioSpec Products Inc., Bartlesville, OK), and homogenized for 60 s at 5,000 rpm in a Mini-BeadBeater (BioSpec Products, Inc., Bartlesville, OK). Eye homogenates were serially diluted and plated in triplicate on BHI agar plates. After overnight incubation at 37°C, the CFU per eye was determined as previously described (52, 53). Values represent mean results ± SEM of at least 8 eyes per group in two independent experiments.

### Statistics.

Reported data are the arithmetic mean results ± the SEM of all samples in the same experimental group in replicate experiments. Statistical significance was set at a *P* level of <0.05. Two-tailed, two-sample *t* tests were used for statistical comparisons between two groups, and one-way analysis of variance was used for comparisons of multiple groups (for the hemolytic assays). The Mann-Whitney U test was used to compare results for experimental groups for the ERG experiments and bacterial counts per eye. All statistical analyses were performed using Prism 6.05 (GraphPad Software, Inc., La Jolla, CA).
